# Foodborne Illness Outbreaks at Retail Food Establishments — National Environmental Assessment Reporting System, 25 State and Local Health Departments, 2017–2019

**DOI:** 10.15585/mmwr.ss7206a1

**Published:** 2023-06-02

**Authors:** Erin D. Moritz, Shideh Delrahim Ebrahim-Zadeh, Beth Wittry, Meghan M. Holst, Bresa Daise, Adria Zern, Tonia Taylor, Adam Kramer, Laura G. Brown

**Affiliations:** ^1^Division of Environmental Health Science and Practice, National Center for Environmental Health, CDC, Atlanta, Georgia; ^2^Oak Ridge Institute for Science and Education, Oak Ridge, Tennessee; ^3^Bureau of Environmental Surveillance and Policy, New York City Department of Health and Mental Hygiene, New York, New York; ^4^Environmental Health Services, Baltimore County Department of Health, Baltimore, Maryland

## Abstract

**Problem/Condition:**

Each year, state and local public health departments report hundreds of foodborne illness outbreaks associated with retail food establishments (e.g., restaurants or caterers) to CDC. Typically, investigations involve epidemiology, laboratory, and environmental health components. Health departments voluntarily report epidemiologic and laboratory data from their foodborne illness outbreak investigations to CDC through the National Outbreak Reporting System (NORS); however, minimal environmental health data from outbreak investigations are reported to NORS. This report summarizes environmental health data collected during outbreak investigations and reported to the National Environmental Assessment Reporting System (NEARS).

**Period Covered:**

2017–2019.

**Description of System:**

In 2014, CDC launched NEARS to complement NORS surveillance and to use these data to enhance prevention efforts. State and local health departments voluntarily enter data from their foodborne illness outbreak investigations of retail food establishments into NEARS. These data include characteristics of foodborne illness outbreaks (e.g., etiologic agent and factors contributing to the outbreak), characteristics of establishments with outbreaks (e.g., number of meals served daily), and food safety policies in these establishments (e.g., ill worker policy requirements). NEARS is the only available data source that collects environmental characteristics of retail establishments with foodborne illness outbreaks.

**Results:**

During 2017–2019, a total of 800 foodborne illness outbreaks associated with 875 retail food establishments were reported to NEARS by 25 state and local health departments. Among outbreaks with a confirmed or suspected agent (555 of 800 [69.4%]), the most common pathogens were norovirus and *Salmonella*, accounting for 47.0% and 18.6% of outbreaks, respectively. Contributing factors were identified in 62.5% of outbreaks. Approximately 40% of outbreaks with identified contributing factors had at least one reported factor associated with food contamination by an ill or infectious food worker. Investigators conducted an interview with an establishment manager in 679 (84.9%) outbreaks. Of the 725 managers interviewed, most (91.7%) said their establishment had a policy requiring food workers to notify their manager when they were ill, and 66.0% also said these policies were written. Only 23.0% said their policy listed all five illness symptoms workers needed to notify managers about (i.e., vomiting, diarrhea, jaundice, sore throat with fever, and lesion with pus). Most (85.5%) said that their establishment had a policy restricting or excluding ill workers from working, and 62.4% said these policies were written. Only 17.8% said their policy listed all five illness symptoms that would require restriction or exclusion from work. Only 16.1% of establishments with outbreaks had policies addressing all four components relating to ill or infectious workers (i.e., policy requires workers to notify a manager when they are ill, policy specifies all five illness symptoms workers need to notify managers about, policy restricts or excludes ill workers from working, and policy specifies all five illness symptoms requiring restriction or exclusion from work).

**Interpretation:**

Norovirus was the most commonly identified cause of outbreaks reported to NEARS, and contamination of food by ill or infectious food workers contributed to approximately 40% of outbreaks with identified contributing factors. These findings are consistent with findings from other national outbreak data sets and highlight the role of ill workers in foodborne illness outbreaks. Although a majority of managers reported their establishment had an ill worker policy, often these policies were missing components intended to reduce foodborne illness risk. Contamination of food by ill or infectious food workers is an important cause of outbreaks; therefore, the content and enforcement of existing policies might need to be re-examined and refined.

**Public Health Action:**

Retail food establishments can reduce viral foodborne illness outbreaks by protecting food from contamination through proper hand hygiene and excluding ill or infectious workers from working. Development and implementation of policies that prevent contamination of food by workers are important to foodborne outbreak reduction. NEARS data can help identify gaps in food safety policies and practices, particularly those concerning ill workers. Future analyses of stratified data linking specific outbreak agents and foods with outbreak contributing factors can help guide the development of effective prevention approaches by describing how establishments’ characteristics and food safety policies and practices relate to foodborne illness outbreaks.

## Introduction

Each year, state, tribal, local, and territorial health departments (hereafter referred to as health departments) report hundreds of foodborne illness outbreaks to CDC (*1*). During 2009–2015, health departments reported 5,760 foodborne illness outbreaks (*2*). A majority of these outbreaks occurred in retail food establishments (e.g., restaurants or caterers), defined as operations that store, prepare, package, serve, or vend food directly to the consumer or otherwise provide food for human consumption (*2*,*3*).

Health departments typically are responsible for regulating and ensuring food safety in retail food establishments, primarily through routine inspections to identify and correct violations of their jurisdictions’ food safety regulations. The U.S. Food and Drug Administration (FDA) Food Code underlies a majority of jurisdictions’ food safety regulations. The FDA Food Code is a model set of science-based, comprehensive food safety recommendations intended to reduce foodborne illness risk in retail food establishments (*4*). For example, the Food Code includes recommendations to limit opportunities for food workers to contaminate food, such as washing hands, using gloves, and prohibiting ill or infectious workers from working with food when they are experiencing specified symptoms. Although the Food Code represents recommendations for food safety, adoption of its provisions, in whole or in part, is voluntary and at the discretion of state and local governments (*4*).

Health departments also are typically responsible for investigating suspected foodborne illness outbreaks in retail food establishments to control and stop the outbreak. Health departments provide epidemiologic and laboratory data from their foodborne outbreak investigations to CDC through the National Outbreak Reporting System (NORS). The data reported include the etiologic agent; food vehicle; outbreak setting; and number of illnesses, hospitalizations, and deaths associated with an outbreak (*5*). These data have led to discoveries of new and emerging foodborne illness agents and specific agent–food pairs (*6*).

In addition to epidemiologic and laboratory data reported to NORS, certain health departments also provide data to CDC from the environmental health component of their investigations, often called the environmental assessment, through the National Environmental Assessment Reporting System (NEARS) (*7*). Since 2014, when NEARS began data collection, 29 health departments have voluntarily reported environmental assessment data from foodborne illness outbreak investigations to NEARS. The data collected describe how the retail food service environment contributes to the introduction or transmission of agents that lead to outbreaks. NEARS collects data on food preparation policies and practices, the processes used in preparing food items suspected in the outbreak, and workers’ food preparation practices (*3*,*8*,*9*). These environmental health data can be used in combination with epidemiologic and laboratory data to gain a comprehensive understanding of an outbreak and identify gaps in the establishments’ food safety policies and practices.

This report summarizes selected data reported to NEARS for foodborne illness outbreaks that occurred during 2017–2019, the most recent years for which final data are available. The findings describe the outbreaks, the establishments where the outbreaks occurred, and food safety policies of those establishments, with an emphasis on policies focused on identifying and managing ill workers. Contamination of food by ill food workers is a top contributing factor to foodborne outbreaks in retail food establishments (*3*,*8*); therefore, identifying gaps in these establishments’ ill worker policies is important to outbreak prevention. Health departments responsible for ensuring food safety in retail food establishments can use the findings in this report to assess their food safety priorities and guide their outbreak investigations and routine (i.e., preventive) inspections.

## Methods

### Description of the System and Case Definition

In 2014, NEARS was launched to collect environmental assessment data during foodborne illness outbreaks associated with retail food establishments (*6*,*7*). CDC defines a foodborne illness outbreak as an incident in which two or more persons experience a similar illness resulting from ingesting a common food (*10*); a majority of health departments have a similar definition. Identified outbreak agents are classified as confirmed if they were laboratory confirmed according to CDC laboratory and clinical guidelines (*10*); otherwise, they are classified as suspected.

### Participating Sites

 For this report, NEARS data were submitted by Alaska; California; Connecticut; Delaware; Fairfax County, Virginia; Georgia; Harris County, Texas; Indiana; Iowa; Jefferson County, Colorado; Kansas City, Missouri; Maricopa County, Arizona; Massachusetts; Michigan; Minnesota; New York; New York City, New York; North Carolina; Oregon; Rhode Island; South Carolina; Southern Nevada Health District; Tennessee; Washington; and Wisconsin. These health departments reported environmental assessment data from at least one foodborne illness outbreak occurring in a retail food establishment.

### Data Sources, Collection, and Availability

Data collected and entered into NEARS are from three sources: observations or determinations made by the environmental health professional conducting the investigation, interviews with the managers of establishments with outbreaks, and the epidemiology or laboratory counterparts at health departments (Box 1). After each foodborne illness outbreak investigation is completed, participating health departments voluntarily report their environmental health investigation data to CDC through the NEARS online data management system on CDC’s website. Not all data elements are collected during all investigations; therefore, denominators vary throughout the results. Data on foodborne illness outbreaks reported to NEARS included in this report are publicly available at https://stacks.cdc.gov/view/cdc/127053.

BOX 1 **Data sources for characteristics of foodborne illness outbreaks and retail food establishments with outbreaks — National Environmental Assessment Reporting System, 25 state and local health departments, 2017–2019**

**Table:** 

**Data collected**	**Data source**
**Outbreak characteristics**	
Primary agent identified as confirmed (i.e., laboratory confirmed by CDC guidelines) or suspected (i.e., not confirmed by guidelines)	Epidemiology and laboratory investigation counterparts
Contributing factor identification (factors that contribute to the contamination, proliferation, and survival of foodborne agents on food)	Investigation team determination*
Outbreak also reported to NORS	Epidemiology and laboratory investigation counterparts
**Outbreak establishment characteristics**	
Ownership: Independent or chain (establishment shares name and operations with at least one other establishment)	Establishment manager interview
Average number of meals served daily	Establishment manager interview
Establishment type: Restaurant (fixed establishment that prepares and serves food) or other (e.g., institutions or restaurants in supermarkets)	Environmental health investigator determination
Most complex food preparation process Complex: Food item requires a kill step (a process, such as cooking or freezing, that reduces pathogens on food) and holding beyond same-day service, or a kill step and a combination of holding, cooling, reheating, and freezing Cook-serve: Food item is prepared for same-day service and requires a kill step Prep-serve: Food item does not require a kill step	Environmental health investigator determination
Menu type (e.g., American or Indian)	Environmental health investigator determination
Number of critical violations on previous inspection (i.e., violations of regulations that help eliminate or reduce hazards associated with foodborne illness; also called priority or priority foundation items)	Environmental health investigator determination
**Outbreak establishment ill worker policies**	
Policy requires workers to tell their manager when they are ill; policy is written	Establishment manager interview
Policy specifies symptoms workers need to tell their manager about (i.e., vomiting, diarrhea, jaundice, sore throat with fever, and lesion with pus)	Establishment manager interview
Policy restricts or excludes ill workers from working; policy is written	Establishment manager interview
Policy specifies symptoms that require restriction or exclusion from work (i.e., vomiting, diarrhea, jaundice, sore throat with fever, and lesion with pus)	Establishment manager interview
Paid sick leave is available for at least one worker	Establishment manager interview
**Abbreviation:** NORS = National Outbreak Reporting System. * Determination of contributing factors is most often a collaborative effort between the environmental health investigators and their epidemiology and laboratory counterparts.	

### Variables Included

For this report, data were collected and presented on three sets of variables: characteristics of foodborne illness outbreaks, characteristics of establishments linked with outbreaks, and ill worker policies of establishments linked with outbreaks.

- **Outbreak characteristics**. Characteristics include the outbreak agent and contributing factors. FDA and CDC have identified three groups of outbreak contributing factors (*11*):
  - contamination of food with a foodborne illness agent,
  - proliferation or growth of microbial agents in food (proliferation can mean an increase in the number of bacteria, the production of toxins, or both), and
  - survival of foodborne illness agents after a process (e.g., cooking) that should have eliminated or reduced them.
- **Outbreak establishment characteristics.** Characteristics include those that have been hypothesized or found to be associated with retail food establishment food safety. These characteristics include ownership (independent or chain, defined in NEARS guidance as an establishment that shares a name and operations with at least one other establishment) and number of meals served daily (*12*–*15*).
- **Outbreak establishment ill worker policies.** Policies assessed include those designed to limit opportunities for food workers to contaminate food by prohibiting workers who are ill or infectious from working with food (*4*). The report also assessed whether these policies were written. The Food Code recommends written plans and procedures (*4*). Specifically, data were presented on whether establishments provided paid sick leave to workers and whether establishments with outbreaks had policies addressing four components of the Food Code relating to ill or infectious workers. This report assessed whether establishments had policies that
  - required workers to tell a manager when they are ill,
  - specified the five symptoms of foodborne illness workers need to report to their manager (i.e., vomiting, diarrhea, jaundice, sore throat with fever, and lesion with pus),
  - restricted (i.e., prevented from handling food) or excluded (i.e., prevented from working) ill or infectious workers, and
  - specified the five symptoms requiring worker restriction or exclusion from work activities.

### Data Analysis

CDC calculated descriptive statistics on characteristics of foodborne illness outbreaks and characteristics and policies of establishments linked with outbreaks. Data cleaning, management, and analysis were conducted using SAS (version 9.4; SAS Institute) and Microsoft Excel for Microsoft 365 MSO (version 2022; Microsoft Corporation). This activity was reviewed by CDC and was conducted consistent with applicable federal law and CDC policy.* 

## Results

During 2017–2019, a total of 800 foodborne illness outbreaks associated with 875 retail food establishments were reported to NEARS by the 25 participating state and local health departments. Among the 800 outbreaks, 216 (27.0%) occurred in 2017, 306 (38.3%) in 2018, and 278 (34.8%) in 2019. Of these outbreaks, 725 (90.6%) involved one establishment and 75 (9.4%) involved multiple establishments. Twenty-eight (3.5%) were multistate outbreaks. Investigators conducted an interview with a manager in 679 (84.9%) outbreaks.

### Outbreak Characteristics

Investigations identified an etiologic agent in 555 (69.4%) outbreaks. Of these agents, 157 (28.3%) were suspected and 398 (71.7%) were confirmed. A majority of identified agents were viral (48.1%) and bacterial (46.8%); parasitic (2.3%) and toxic or chemical (2.5%) agents accounted for the remainder. The most common agent was norovirus, accounting for 47.0% (65.1% of which were laboratory confirmed), followed by *Salmonella*, accounting for 18.6% (87.4% of which were laboratory confirmed) (Table 1).

**TABLE 1:** Foodborne illness outbreaks with a suspected or confirmed identified agent — National Environmental Assessment Reporting System, 25 state and local health departments, 2017–2019

Agent	Suspected No. (%)*	Confirmed No. (%)*	Total No. (%)*
**Virus **			
Norovirus	91 (16.4)	170 (30.6)	**261 (47.0)**
Hepatitis A	0 (—)	3 (0.5)	**3 (0.5)**
Sapovirus	1 (0.2)	2 (0.4)	**3 (0.5)**
**Total viral outbreaks**	**92 (16.6)**	**175 (31.5)**	**267 (48.1)**
**Bacteria**			
*Salmonella* species	13 (2.3)	90 (16.2)	**103 (18.6)**
*Vibrio* species	5 (0.9)	34 (6.1)	**39 (7.0)**
*Clostridium perfringens*	20 (3.6)	16 (2.9)	**36 (6.5)**
*Campylobacter* species	3 (0.5)	22 (4.0)	**25 (4.5)**
*Escherichia coli,* O157: H7	0 (—)	14 (2.5)	**14 (2.5)**
*Escherichia coli,* other Shiga toxin–producing or verotoxin-producing	1 (0.2)	9 (1.6)	**10 (1.8)**
*Escherichia coli,* other enteric pathogenic	0 (—)	4 (0.7)	**4 (0.7)**
*Shigella* species	0 (—)	9 (1.6)	**9 (1.6)**
Other bacteria	5 (0.9)	1 (0.2)	**6 (1.1)**
*Bacillus cereus*	5 (0.9)	2 (0.4)	**7 (1.3)**
*Staphylococcus aureus*	4 (0.7)	2 (0.4)	**6 (1.1)**
*Listeria monocytogenes*	0 (—)	1 (0.2)	**1 (0.2)**
**Total bacterial outbreaks**	**56 (10.1)**	**204 (36.8)**	**260 (46.8)**
**Parasite **			
*Cyclospora cayetanensis*	2 (0.4)	9 (1.6)	**11 (2.0)**
*Cryptosporidium* species	0 (—)	1 (0.2)	**1 (0.2)**
*Giardia duodenalis*	0 (—)	1 (0.2)	**1 (0.2)**
**Total parasitic outbreaks**	**2 (0.4)**	**11 (2.0)**	**13 (2.3)**
**Toxin or chemical**			
Scombroid toxin or histamine	5 (0.9)	6 (1.1)	**11 (2.0)**
Toxic agent	1 (0.2)	0 (—)	**1 (0.2)**
Chemical agent	1 (0.2)	1 (0.2)	**2 (0.4)**
**Total toxin or chemical outbreaks**	**7 (1.3)**	**7 (1.3)**	**14 (2.5)**
**Multiagent^†^**			
**Total multiagent outbreaks**	**0 (—)**	**1 (0.2)**	**1 (0.2)**
**Total outbreaks**	**157 (28.3)**	**398 (71.7)**	**555 (100.0)**

* Denominator for all percentages is the number of outbreaks with a suspected or confirmed agent (n = 555). Percentages might not total 100% due to rounding.

^†^ Norovirus and *Escherichia coli*.

Investigators identified at least one contributing factor in 500 (62.5%) outbreaks. Outbreaks can have more than one contributing factor, and 819 contributing factors were identified altogether. Of the 500 outbreaks with an identified contributing factor, 426 (85.2%) had at least one contamination factor, 129 (25.8%) had at least one proliferation factor (i.e., conditions allowed pathogens in food to grow), and 71 (14.2%) had at least one survival factor (i.e., pathogens survived processes designed to kill or reduce their numbers) (Table 2).

**TABLE 2:** Factors contributing to foodborne illness outbreaks, overall and by type of factor — National Environmental Assessment Reporting System, 25 state and local health departments, 2017–2019

Contributing factor	No. of outbreaks with this contributing factor	% of outbreaks with a contributing factor in the specific category (contamination, proliferation, survival)*	% of all outbreaks with a contributing factor (denominator = 500)*
**Contamination of food with a foodborne illness agent (denominator = 426 outbreaks with a contamination contributing factor)**			
Other mode of contamination (excluding cross-contamination) by a food handler, worker, or preparer who was suspected to have an infectious illness (C12)^†^	104	24.4	20.8
Contaminated raw product (food was intended to be consumed raw or undercooked or underprocessed) (C7)	88	20.7	17.6
Bare-hand contact of RTE food by a food handler, worker, or preparer who was suspected to have an infectious illness (C10)	72	16.9	14.4
Cross-contamination of ingredients (C9)	68	16.0	13.6
Other source of contamination (C15)	62	14.6	12.4
Gloved-hand contact of RTE food by a food handler, worker, or preparer who was suspected to have an infectious illness (C11)	53	12.4	10.6
Contaminated raw product (food was intended to be consumed after a kill step) (C6)	27	6.3	5.4
Storage in contaminated environment (C14)	15	3.5	3.0
Toxic substance part of the tissue (e.g., ciguatera) (C1)	14	3.3	2.8
Foods contaminated by nonfood handler, worker, or preparer who was suspected to have an infectious illness (C13)	13	3.1	2.6
Foods originating from sources shown to be contaminated or polluted (C8)	12	2.8	2.4
Poisonous substance accidentally or inadvertently added (C3)	3	0.7	0.6
Addition of excessive quantities of ingredients that are toxic in large amounts (e.g., niacin poisoning from bread) (C4)	1	0.2	0.2
**Proliferation or growth of microbial agents in food (increase in number of bacteria or the production of toxins) (denominator = 129 outbreaks with a proliferation contributing factor)**			
Improper or slow cooling (P8)	53	41.1	10.6
Improper cold holding due to malfunctioning refrigeration equipment (P4)	33	25.6	6.6
Food preparation practices that support proliferation of pathogens (during food preparation) (P1)	27	20.9	5.4
Improper hot holding due to an improper procedure or protocol (P7)	25	19.4	5.0
Improper cold holding due to an improper procedure or protocol (P5)	23	17.8	4.6
No attempt to control the temperature of implicated food or the length of time food was out of temperature control (during food service or display of food) (P2)	21	16.3	4.2
Improper adherence to approved plan for using time as a public health control (P3)	12	9.3	2.4
Other situations that promoted or allowed microbial growth or toxin production (P12)	9	7.0	1.8
Prolonged cold storage (P9)	3	2.3	0.6
Improper hot holding due to malfunctioning equipment (P6)	2	1.6	0.4
Inadequate modified atmosphere packaging (P10)	1	0.8	0.2
**Survival of foodborne illness agents after a process, such as cooking, that should have eliminated or reduced them (denominator = 71 outbreaks with a survival contributing factor)**			
Insufficient time, temperature, or both during cooking or heat processing (e.g., roasted poultry, canned foods, or pasteurization) (S1)	33	46.5	6.6
Insufficient time, temperature, or both during reheating (S2)	18	25.4	3.6
Insufficient or improper use of chemical processes designed for pathogen destruction (S4)	17	23.9	3.4
Other process failures that permit agent survival (S5)	10	14.1	2.0

**Source:** CDC [Internet]. Contributing factor definitions 2009–2021. Atlanta, GA: US Department of Health and Human Services, CDC; 2022. https://www.cdc.gov/nceh/ehs/nears/cf-definitions-2009-2021.htm

**Abbreviations:** C = contamination; P = proliferation; RTE = ready-to-eat; S = survival.

* Certain outbreaks had more than one identified contributing factor; thus, percentages can sum to >100%.

^†^ These designations (e.g., C1, P6, and S2) are used by outbreak investigators to refer to the type of contributing factor (e.g., C, P, or S) and its numerical position on the contributing factor list.

The top five contributing factors to foodborne illness outbreaks were all contamination related (Box 2). The most common contributing factor was other mode of contamination (excluding cross-contamination) by a worker who was suspected to have an infectious illness (104 [20.8%]). Other sources of contamination included contaminated raw food (88 [17.6%]), bare-hand contact with ready-to-eat (RTE) food by a food worker suspected to have an infectious illness (72 [14.4%]), cross-contamination of ingredients (68 [13.6%]), and other unspecified source of contamination (62 [12.4%]) (Table 2). Contributing factors associated with ill workers (i.e., bare-hand contact with RTE food, gloved-hand contact with RTE food, and other contamination by workers suspected of having an infectious illness) were identified in 205 (41.0%) outbreaks. The most common proliferation contributing factor was improper or slow cooling of hot food (53 [10.6%]), and the most common survival contributing factor was insufficient time or temperature during cooking or heat processing (33 [6.6%]).

BOX 2 **Top five contributing factors to foodborne illness outbreaks in retail food establishments — National Environmental Assessment Reporting System, 25 state and local health departments, 2017–2019**

**Contributing factor**

1. Other mode of contamination (excluding cross-contamination) by a food handler, worker, or preparer who was suspected to have an infectious illness (C12)*

2. Contaminated raw product (food was intended to be consumed raw, undercooked, or underprocessed) (C7)

3. Bare-hand contact with RTE food by a food worker who was suspected to have an infectious illness (C10)

4. Cross-contamination of ingredients (C9)

5. Unspecified source of contamination (C15)

**Source:** CDC [Internet]. Contributing factor definitions 2009–2021. Atlanta, GA: US Department of Health and Human Services, CDC; 2022. https://www.cdc.gov/nceh/ehs/nears/cf-definitions-2009-2021.htm

**Abbreviations:** C = contamination; P = proliferation; RTE = ready-to-eat; S = survival.

^*^ The designations (e.g., C12) are used by outbreak investigators to refer to the type of contributing factor (e.g., C, P, or S) and its numerical position on the contributing factor list.

### Outbreak Establishment Characteristics

A majority of establishments with outbreaks were independently owned (473 of 725 [65.2%]) and served ≤300 meals (upper range = 8,500 meals) daily (440 of 725 [60.7%]) (Table 3). Most were restaurants (712 of 875 [81.4%]), and 84.0% (735 of 875) served complex food items. Complex food items require a kill step (i.e., a process, such as cooking, that reduces or eliminates foodborne illness pathogens) and holding beyond same-day service, or a kill step and a combination of holding, cooling, reheating, and freezing. The most common menu type was American (485 of 875 [55.4%]). A majority (624 of 875 [71.3%]) of establishments received at least one critical violation on their last routine inspection before the outbreak.

**TABLE 3:** Characteristics of retail establishments with foodborne illness outbreaks — National Environmental Assessment Reporting System, 25 state and local health departments, 2017–2019

Characteristic	No. (%)
Ownership*	
Independent	473 (65.2)
Chain	243 (33.5)
Unsure	9 (1.2)
**Total**	**725 (100.0)**
Number of meals served daily*	
≤100	176 (24.3)
101–200	155 (21.4)
201–300	109 (15.0)
301–400	50 (6.9)
401–500	57 (7.9)
501–8,500	100 (13.8)
Unsure	78 (10.8)
**Total**	**725 (100.0)**
**Establishment type^†^**	
Restaurant	712 (81.4)
Other (e.g., caterer or mobile food unit)	163 (18.6)
**Total**	**875 (100.0)**
**Most complex food preparation process^†^**	
Complex	735 (84.0)
Cook-serve	104 (11.9)
Prep-serve	36 (4.1)
**Total**	**875 (100.0)**
Menu*	
American	485 (55.4)
Mexican	141 (16.1)
Other (e.g., Greek or Hawaiian)	116 (13.3)
Italian	55 (6.3)
Chinese	37 (4.2)
Japanese	32 (3.7)
Thai	9 (1.0)
**Total**	**875 (100.0)**
**Number of critical violations on last inspection^†^**	
None	244 (27.9)
1	191 (21.8)
2	134 (15.3)
≥3	299 (34.2)
Unsure	7 (0.8)
**Total**	**875 (100.0)**

* These data were collected through an interview with the establishment manager; the denominator is the number of establishments in which a manager interview was conducted. Percentages might not total 100% due to rounding.

^†^ These data were reported by the environmental health investigator; the denominator is the number of establishments associated with outbreaks reported to the National Environmental Assessment Reporting System.

### Outbreak Establishment Policies

Most managers interviewed (665 of 725 [91.7%]) said their establishment had a policy requiring food workers to notify their manager when they were ill, and the policy was written (439 of 665 [66.0%]) (Table 4). Approximately 75% (504 of 665 [75.8%]) had policies that required ill food workers to tell managers their symptoms; 452 (68.0%) specified vomiting or diarrhea (each) as symptoms workers needed to tell managers about. Fewer policies mentioned sore throat with fever (328 [49.3%]), lesion with pus (265 [39.8%]), and jaundice (182 [27.4%]). Only 23.0% (153) of policies listed all five symptoms workers needed to tell managers about.

**TABLE 4:** Manager interview data on policies requiring food workers to tell managers when they are ill, for retail establishments with foodborne illness outbreaks — National Environmental Assessment Reporting System, 25 state and local health departments, 2017–2019

Policy	No. (%)
**Establishment has a policy that requires food workers to tell a manager when they are ill**	
Yes	665 (91.7)
No	45 (6.2)
Unsure	12 (1.7)
Refused	3 (0.4)
**Total**	**725 (100.0)**
**For establishments with a policy that requires food workers to tell a manager when they are ill**	
The policy requiring workers to tell a manager when they are ill is in writing*	
Yes	439 (66.0)
No	203 (30.5)
Unsure	23 (3.5)
**Total**	**665 (100.0)**
The policy requires ill workers to tell a manager about their symptoms*	
Yes	504 (75.8)
No	132 (19.8)
Unsure	29 (4.4)
**Total**	**665 (100.0)**
The policy specifies the symptoms that workers are to tell managers about*	
Vomiting	452 (68.0)
Diarrhea	452 (68.0)
Jaundice	182 (27.4)
Sore throat with fever	328 (49.3)
Lesion with pus	265 (39.8)
**Total**	**665 (100.0)^†^**

* These questions are asked only of managers who said their establishment had a policy requiring food workers to tell managers when they are ill.

^†^ This question was an open-ended question, and the 665 managers who responded could mention multiple symptoms; thus, the percentages can sum to >100%.

Of the managers interviewed, most (620 of 725 [85.5%]) said that their establishment also had a policy restricting or excluding ill food workers from working, and these policies were written (387 of 620 [62.4%) (Table 5). A majority (431 of 620 [69.5%]) said these policies specified symptoms that would prompt restriction or exclusion. Nearly two thirds of policies specifically mentioned vomiting (406 [65.5%]) and diarrhea (410 [66.1%]) as symptoms that would require restriction or exclusion. Fewer policies mentioned sore throat with fever (283 [45.6%]), lesion with pus (231 [37.3%]), and jaundice (165 [26.6%]). Only 17.8% (129) of policies listed all five symptoms that would require restriction or exclusion.

**TABLE 5:** Manager interview data on policies restricting* or excluding ill food workers from working, for retail establishments with foodborne illness outbreaks — National Environmental Assessment Reporting System, 25 state and local health departments, 2017–2019

Policy	No. (%)
**The establishment has a policy to restrict or exclude ill food workers from working**	
Yes	620 (85.5)
No	77 (10.6)
Unsure	27 (3.7)
Refused	1 (0.1)
**Total**	**725 (100.0)**
**For establishments with a policy to restrict or exclude ill food workers from working**	
**The policy to restrict or exclude ill workers is in writing^†^**	
Yes	387 (62.4)
No	200 (32.3)
Unsure	33 (5.3)
**Total**	**620 (100.0)**
**The policy specifies the symptoms that would prompt restricting or excluding ill workers from working^†^**	
Yes	431 (69.5)
No	149 (24.0)
Unsure	40 (6.5)
**Total**	**620 (100.0)**
**Symptoms specified in the policy that require restriction or exclusion from work** ^†^	
Vomiting	406 (65.5)
Diarrhea	410 (66.1)
Jaundice	165 (26.6)
Sore throat with fever	283 (45.6)
Lesion with pus	231 (37.3)
**Total**	**620 (100.0)^§^**

* Restrict means to limit the activities of a food worker so that there is no risk for transmitting a disease that is transmissible through food, and the food worker does not work with exposed food, clean equipment, utensils, linens, or unwrapped single-service or single-use articles (**Source:** Food and Drug Administration. Food code. Washington, DC: US Department of Health and Human Services, Public Health Service, Food and Drug Administration; 2017. https://www.fda.gov/media/110822/download).

^†^ These questions are asked only of managers who said their establishment had a policy to restrict or exclude ill workers from working.

^§^ This question was an open-ended question, and the 620 managers who responded could mention multiple symptoms; thus, the percentages can sum to >100%.

Only 16.1% (117 of 725) of establishments had policies that included the four recommendations of the FDA Food Code that were assessed. These recommendations were to have a policy that required workers to tell a manager when they are ill, a policy that specified all five symptoms workers need to tell a manager about, a policy that restricted or excluded ill or infectious workers from working, and a policy that specified all five symptoms requiring restriction or exclusion. Fewer than half (316 of 725 [43.6%]) of managers said their establishments provided paid sick leave to any workers.

##  Discussion

During 2017–2019, norovirus was the most common cause of outbreaks in retail food establishments reported to NEARS, and the most common contributing factor was “other” contamination by a food worker suspected to have an infectious illness. Examples include non–cross-contamination sources such as aerosolized vomitus and outbreaks where investigators could not determine if the food worker was wearing gloves during food preparation. At least one of three contributing factors associated with suspected infectious workers (bare-hand contact with RTE food, gloved-hand contact with RTE food, and other contamination by a suspected infectious worker) was reported in 41.0% of the outbreaks. These findings are similar to those from the 2014–2016 NEARS surveillance period and in national outbreak data reported to NORS (*3*).

One way to help prevent foodborne illness in retail food establishments is to adopt comprehensive food safety policies. Such policies have been linked to improved food safety outcomes (e.g., increased frequency of equipment cleaning and proper date marking) (*16*). Establishment policies might also mitigate the size of outbreaks. Outbreak establishments with cleaning and glove use policies had smaller norovirus outbreaks than those without such policies. Moreover, the form of the policy was associated with outbreak size; outbreak establishments with written policies had smaller outbreaks than those with only verbally communicated policies (*17*).

Ill workers continue to play a substantial role in retail food establishment outbreaks (*3*,*8*,*16*), and comprehensive ill worker policies will likely be necessary to mitigate this public health problem. Restaurants with policies requiring workers to report illness to managers were less likely to have employees who worked while ill (*18*). Most outbreak establishments with manager interview data had written or verbally communicated policies requiring ill workers to tell managers when they were ill (91.7%) and restricting or excluding ill workers (85.5%) from working. However, managers indicated that their ill worker policies did not include all of the five symptoms of illness itemized in the FDA Food Code (i.e., vomiting, diarrhea, jaundice, sore throat with fever, and lesion with pus). Vomiting and diarrhea, two of the most common symptoms of foodborne illness, were specified most often (range = 65.5%–66.1% of establishments with outbreaks). However, approximately one third of establishments did not specify these two symptoms. Policies might need to be comprehensive to be effective; only 16.1% of outbreak establishments with manager interview data had all four components of ill worker policies that were assessed.

Although research suggests that written policies are more effective than verbally communicated policies (*17*), the existence of written policies alone is unlikely to markedly reduce incidence of foodborne illness outbreaks in retail establishments. Policy implementation and compliance are also important. Recent FDA modeling data indicated that high compliance with policies excluding ill food employees substantially decreased predicted illnesses (*19*). Moreover, policies that are regulatory requirements might have greater likelihood of effectiveness. For example, states with a regulatory requirement to exclude ill employees from working had lower norovirus outbreak rates than states without this requirement (*20*). A lack of regulatory requirements might reduce the likelihood of officials thoroughly assessing policy components during inspections. In contrast, policies assessed during inspections are likely prioritized for implementation and enforcement.

Food workers report numerous reasons for working when ill, such as loss of pay and perceived social pressure (*18*). NEARS data demonstrate that fewer than half of establishments with outbreaks provided paid sick leave to at least one food worker. Research suggests that paid sick leave might improve food safety outcomes. Expanded paid sick leave in a restaurant chain reduced the incidence of working while ill among front-line food service workers (*21*), and supportive paid sick leave regulations were found to be associated with decreased foodborne illness rates (*22*). A multilayered approach addressing implementation and enforcement might be required to prevent ill employees from working. Such an approach not only includes adoption and enforcement of comprehensive written ill worker policies but also enhances training, management plans to continue operations when a worker is absent (e.g., on-call staffing), and adoption of a food safety culture where absenteeism due to illness is not penalized (*17*,*18*,*23*).

Approximately half of the outbreaks reported to NEARS were caused by a bacteria, including *Salmonella,* that either exist at unsafe levels in foods (e.g., *Escherichia coli* O157:H7 in ground beef) or have contaminated food at a certain point in the food production chain. Bacteria on food can be eliminated or reduced through a kill step (e.g., cooking). However, if contaminated food does not go through a kill step or the kill step is inadequate (e.g., undercooking), the bacteria can survive and proliferate, particularly when the food is not maintained at adequate temperatures. Moreover, the majority of establishments with outbreaks engaged in complex processes that might have increased the likelihood of pathogen proliferation or survival because these processes involve riskier food preparation practices (e.g., reheating, cooling, and holding). Taken together, these findings are a reminder that following Food Code guidance on cross-contamination prevention and proper cooking, reheating, holding, and cooling of food is important to prevent bacterial illness (*4*).

## Limitations

The findings in this report are subject to at least six limitations. First, data are reported voluntarily by a limited number of state and local health departments. Although these health departments represent geographically diverse areas, the foodborne illness outbreaks reported to NEARS might not be representative of all U.S. outbreaks. Second, not all outbreaks are identified, reported, or investigated; therefore, the extent to which the outbreaks reported to NEARS represent all outbreaks that occurred in the reporting areas is unknown. Third, outbreak investigation procedures and practices vary across state and local health departments, possibly resulting in systematic differences in data collection. Fourth, manager interview data were based on managers’ recall of policies and practices. For example, managers were asked to list symptoms in their establishments’ ill worker policies from memory. Although this interview method was chosen so that findings were more reflective of conditions and practices in the establishment, written policies in place might have been more comprehensive than captured in the data. Fifth, manager interviews might also be subject to social desirability bias, in which respondents overreport socially desirable conditions (e.g., the existence of food safety policies in their establishments). Finally, these data were collected before the COVID-19 pandemic. Evidence suggests that retail food establishments changed at least some of their practices during the pandemic (*21*), and certain changes might be permanent. Thus, the data reported might not be representative of current practices.

## Future Directions

Future NEARS analyses will focus on stratifying data by etiologic agent to identify the contributing factors of outbreaks linked with specific agents (e.g., *Salmonella*) and foods (e.g., poultry and vegetables). Regression modeling can be used to assess risk factors associated with specific agents. Root cause analyses of norovirus outbreaks, in particular, might be useful in identifying policies and practices to reduce outbreaks associated with retail establishments. Future analyses also will identify longitudinal trends in NEARS data, such as whether the percentage of establishments with outbreaks that have comprehensive ill worker policies has changed since 2014 when NEARS was launched. Finally, matching NEARS environmental health data with NORS epidemiologic and laboratory data will enable the examination of associations between establishment policies and practices and outbreak size. An analysis of this type found that the existence of certain food safety policies in establishments with outbreaks (environmental health data) were linked with smaller norovirus outbreaks (epidemiologic data) (*17*). These types of findings can help guide and develop outbreak prevention efforts.

## Conclusion

NEARS provides important environmental data on retail food establishments that have had foodborne illness outbreaks. These data increase knowledge about the environmental context of outbreaks and contribute to generating and testing hypotheses about outbreak causes and prevention. The analyses identified primary contributing factors to outbreaks and gaps in establishment policies related to ill workers. The findings in this report can help public health authorities and the retail food establishment industry develop data-driven, effective approaches to preventing foodborne illness outbreaks (*7*).
